# Surgical Treatment of Aneurism

**Published:** 1874-06

**Authors:** Timothy Holmes


The Surgical Treatment of Aneurism.—*By Timothy Holmes.*—I hope the facts I have brought under your notice have been sufficient to establish the following propositions with respect to axillary aneurism:—

1. That there are a great number of these aneurisms, both traumatic and spontaneous, which are amenable to gradual, intermitting pressure, when carefully applied to the artery above the tumour.

2. That in cases where this is not possible, from the pain which the patient experiences on pressure, the application of rapid total compression, under anæsthesia, may effect a cure.

3. That the ligature of the subclavian artery is so dangerous an operation, both from its own risks and from the proximity of the sac, that it ought to be restricted to cases where pressure has failed, and to those in which, from the size and rapid growth of the axillary tumour, the surgeon thinks pressure unadvisable.

4. That the old operation is to be preferred to the ligature of the subclavian in cases of ruptured artery; and that it may be practiced in cases where, from the elevation of the shoulder or from the extent of the tumour, the surgeon would find it difficult to tie the subclavian, or fears in doing so to injure the sac; but that the anatomical relations of axillary aneurism render this a peculiarly hazardous proceeding, and the surgeon should always be prepared to amputate if necessary.

